# Nasal Polypus

**Published:** 1867-02

**Authors:** Charles B. S. Davis

**Affiliations:** Meridan, Conn.


ART. II.—*Nasal Polypus.* By CHARLES B. S. DAVIS, M. D., *Meridan, Conn.*

Of the various affections to which the nose is liable, none are more distressing than nasal polypus. Polypus may originate in any of the mucous canals, and are found in the uterus, antrum, rectum, larynx, and meatus auditorius. They occur at all periods of life, but are most frequent about middle age. Mr. Pott has taken pains to show that there is one kind of polypus originally *benign;* another originally *malignant.* [1] Mr. John Bell contends that a polypus is never mild, and never malignant; but that time and the natural growth of the tumor, and the pressure it occasions within the soft and bony cells of the nostrils and jaws, must bring every polypus to one invariable form, in its last and fatal stage. Every polypus, according to Mr. Bell, in its early stage, is a small, movable tumor, attended with a sneezing and watering of the eyes; swelling in moist weather, descending with the breath, but easily repressed with the point of the finger. Yet this little tumor continues to grow, the bones become absorbed, the membranes ulcerate, the teeth fall from the sockets, and, through the empty socket, a foul and fetid matter issues from the antrum. Mr. Bell observes "that if horrid symptoms could establish the fact of malignity, there is not to be found in all nosology a more malignant disease than this." [2] I shall proceed to consider three varieties of polypi of the nose.

I.—*The common gelatinous polypus.* This is a tumor of the consistence of jelly, pear-shaped; some being fleshy and of a pale color, while others are of a yellowish tint, and are attached by a narrow neck to the mucous membrane, where it covers the upper part of the nostrils, sometimes where it lies upon the inferior spongy bone, but are rarely if ever attached to the septum. They sometimes grow so far back in the posterior nares, as to pass but slightly into the nose, but project backwards into the pharynx, to some part of which they may be adherent. One nostril is rarely affected alone. They apparently consist of organized lymph, and enlarge until the cavity is filled, and then remain stationary, though sometimes if permitted to remain it continually increases in size, blocks up the nostrils, displaces the septum, and obstructs the other nostril, and causes sometimes eformity of the cheek, and even death by pressure on the brain. The patient has a constant feeling of stuffing and cold in the head, dullness or total loss of smell and taste, and sometimes deafness. It is generally observed that the patient is worse in a warm room, and in damp weather, than at other times.

2.—*The hydated polypus.* This is a rare species, and is described by Druitt as consisting of a number of thin vesicles filled with a watery fluid, and attached by a peduncle. The vesicles burst upon the slightest pressure, and their re-production may be prevented by touching the peduncle frequently with a hair-pencil dipped in butter of antimony. [3]

3.—*The malignant, carcinomatous or fungoid polypus.* The subjects of this form of the disease, manifest so distinctly cachectic diathesis, that it is easily diagnosed. It is generally accompanied from the commencement with pain in the nose and forehead and general headache. It is not affected by change of weather or other circumstance that influences the mild form of the disease. It has a peculiarly firm consistence, is of a red color, some pain is felt on pressure, and bleeding is easily produced. This polypus may go on slowly increasing for years, until it ends in a cancerous or fungus disease, and sometimes produces a frightful destruction of all the neighboring parts. Long before this, however, the skin over it assumes the liver color of the polypus itself. This form of polypus never becomes mild. Colles is of the opinion that it should not be meddled with, and cites a case where the patient died in twenty-four hours after an attempt had been made to extract it. [4] But in the opinion of Richter, neither the malignant nature of a polypus, its adhesions, immoveableness, ulceration, nor disposition to hemorrhage, are any apology for leaving the disease to itself. Ferguson mentions a case where the tumor had been of several years' growth, and projected prominently on the face on each side of the nose. In each nostril a large mass could be seen, and a probe could be passed with ease below and on both the outer sides; but as doubts were entertained regarding the attachments in the middle and above, it was not considered advisable to resort to any operation. Some weeks after the patient died, and the post-mortem examination showed, that the tumor extended from the ethmoid bone to the condyles of the occipital, and was also attached to both sides of the septum. Two large pendulous bodies hung down into the pharynx. The turbinated bones were absorbed, but the mucous membrane which contained them was entire. There was no attachment to the outer walls of the nasal cavity. There was a large abscess in the left anterior lobe of the brain, with an opening leading from it to the nose. [5] Cooper mentions the case of a boy in St. Bartholomew's Hospital. The tumor before death had expanded the upper part of the nose to an enormous size, while below, the nostril was immensely enlarged. The distance between the eyes was extraordinary, being more than four inches. The left eye was affected with amaurosis, brought on by the pressure of the swelling; the right retained to the last the faculty of seeing. The tumor nearly covered the mouth, so that food could only be introduced with a spoon, and an examination of the state of the palate was impossible. On examination of the head after death, a good deal of the tumor was found to be of a cartilaginous consistence, and what was most remarkable, a portion of it, which was as large as an orange, extended within the cranium, where it had annihilated the anterior lobe of the left hemisphere of the brain. Yet, notwithstanding this effect, the boy was not comatose, nor insensible, till a few hours before his death. All the surrounding bones had been more or less absorbed, and the place from which the excrescence first grew, could not be determined. [6]

Treatment.—Says M. Pott, "The polypus is a disease which, of all others, is said to be most difficult totally and perfectly to eradicate, and most liable to re-production." There are four modes of extirpating a nasal polypus, viz: extracting it with forceps; tying it with a ligature; cutting it out; and destroying it with caustic.

1.—*Extracting it with forceps.* This method is mentioned in the works that Sprengel ascribes to Thessalus and Dracon, the son of Hippocrates. Brunus recommends that we should remove the fleshy excrescences by means of a hook, and G. de Salicet had already advised the forceps. We are indebted to Dionis for the first circumstantial details upon the subject of employing them methodically. Since adopted by almost all practitioners, they have been modified by Sharpe, who sometimes employed such as were curved; by B. Bell, who pierced or made an aperture through their branches, and by Richtel, who for large sized polypi invented a kind whose branches could be adjusted separately. The straight forceps are the best whenever the situation of the tumor allows of their application. The patient should be seated opposite to a window, and made to expire strongly; then the forceps a little opened should be introduced to the highest point, and the polypus should be grasped as near its attachment as possible; the forceps should then be twisted round and round slowly, drawing them steadily downwards, but not too strongly, until the resistance gives way and the polypus comes out. Sometimes it is necessary to introduce the forceps several times and remove the substance piecemeal. When the tumor extends beyond the posterior opening of the nasal fossa, it is rarely practicable to extract it entire through the nose. It is under such circumstances that curved hooks became indispensable for seizing it through the pharynx. The tearing out of polypi is rarely followed by serious accidents. Hemorrhage generally succeeds the operation, but not to any serious extent, and is easily controlled. The patient should be informed that the polypus is very likely to grow again. This may be owing to some of the roots having been left behind; from some constitutional causes, from some local morbid affection of the Schneiderian membrane, or of the bones situated beneath the root of the polypus.

2.—*The use of the ligature.* The ligature goes back to the remotest antiquity, but we have to come down to the 16th and 17th centuries before we find it clearly described. Fallopius applied it by means of a brass wire, a noose of which he directed around the polypus by means of a silver canula. Glandorp, who paid particular attention to it, applied the ligature by means of a kind of needle shaped lik a hook, and having an eye near its point, which was furnished with a silk thread. Levret proposed to conduct a silver wire by means of a sound upon the root of the tumor, to make its two extremities afterwards pass through a double canula, in order to twist them by turning it on its axis, after having fastened them to the rings which it has on its free extremity. Different methods of applying the ligature have been introduced; those of Brasdor, Desault, Dubois, Rigand and Hatin, are the most noted. The wire should be of the purest silver and very flexible, that it may not readily break. The noose is to be applied in the following manner:—The polypus should be drawn down as far as possible, by means of the forceps, the noose is then to be carried over the forceps and polypus into the nostril, and pushed up as far as possible; as soon as the noose has been introduced as deeply as possible, the loose end of the wire is to be drawn out of the lower aperture of the canula, and rolled around the ring on that side of the instrument; this must be tightened from day to day, until the separation is effected. The ligature seldom accomplishes an entire destruction of the disease, as it is generally impracticable to introduce the noose to a sufficient depth to include the root.

3.—*Excision.* The use of cutting instruments has always been reprobated in the treatment of polypus, because they usually occasion a profuse hemorrhage, and can hardly ever be passed, without mischief, to a sufficient depth into the nose to divide the root of the tumer. Yet there are instances in which they can be used with advantage. Hippocrates appears to have used cutting instruments in the treatment of polypus, and Celsus mentions a species of cutting-plate, (*spatha*) by which it was practiced. Paul cut out the polypus by means of his *spathapolypica*, one of the extremities of which had attached to it a chisel, and tore out the rest by a *polypoxista.* Scacchi operated with the simple bistoury. Hutten, with a syringotome; Nessi, with a curved and blunt pointed bistoury. J. Fabricus warmly extols a kind of forceps in the shape of a double cutting spoon, which M. A. Severin accuses him of having borrowed from Nicollini, without acknowledging it; an instrument which Glandorp, V. Horne and Solingen has successively modified, and which Dionis, Percy and B. Bell have considered should not be entirely rejected from practice. [7]

4.—*Caustics.* The cautery which was formerly extensively used for the cure of nasal polypi, is now almost entirely rejected. Hippocrates was in favor of the hot iron; Philoxenes, according to Galen, preferred arsenic, acetate and sulphate of copper; while Antipater and Masa also made use of vermilion of Sinape. These different articles were applied by means of tents or rolls of lint, pieces of sheet-lead, metallic tubes, etc., in order to touch its prominent part and destroy it by degrees. Afterwards they substituted for these, injections of lime-water, solutions of alum or vitriol, astringent or styptic decoctions, finally the whole array of desiccative medicaments. A German empiric by the name of Jensch, acquired considerable notoriety in the cure of obstinate polypi, by the use of sulphuric acid, butter of antimony and nitrate of silver. M. Wagner, in 1827, published some remarkable cases treated by this remedy. In employing the actual cautery, says Richter, the object is not to effect, by its direct agency, a gradual destruction of the polypus, but to excite such an inflammation and suppuration of the whole of it, as shall lead to this event. In Newton's edition of Syme's Surgery, the local application of proper caustics and tonic astringents, is recommended as of more importance than the mere removal of the developed polypus; and for the purposes of cauterization the sulphate and chloride of zinc are recommended.

In the words of Mr. Pott, "I cannot leave this subject without cautioning the young practitioner to be exceedingly careful in examining and inquiring into all the circumstances previous to his undertaking a cure, lest he should find, too late, that he has gone too far to recede. For want of such caution, I have seen hemorrhages which have been frightful, and inflammations which have proved fatal. I have seen a case wherein an untoward looking polypus, and which ought not to have been meddled with, has been so attached to a distempered septum nasi, that it has come away with it. I have seen the same thing happen with regard to almost the whole of the ossa palati; and I have more than once known a polypus thickening of the membrane covering the ossa spongiosa, and septum nasi, which, in all probability, would have remained quiet a great length of time, so irritated by rough treatment and unsuccessful attempts, as to render the remainder of the patient's life truly miserable to himself and offensive to others." [8]

1. Chirurgical Works, vol. iii, p. 209.
2. Principles of Surgery, vol. iii, p. 90.
3. Principles and Practice of Modern Surgery. p. 383.
4. Lectures on the Theory and Practice of Surgery, p. 108.
5. Practical Surgery, p. 491.
6. Surgical Dictionary, Art. Polypus.
7. Velpeau's Operative Surgery, vol. iii, p. 406.
8. Chirurgical Works, vol, iii, p. 223.

